# Public Speaking

**Published:** 1873-12

**Authors:** 


### PUBLIC SPEAKING.

A MAN can speak easier when he is full of his subject than when he has little or no heart in it.

A man can speak easier if his audience is intensely interested than if they must be worked up to the subject. To interest an audience the very first necessity is

### ENUNCIATION.

So speak that every word shall be clearly, fully, and so distinctly spoken that it requires no effort to hear it; for, in proportion as it does, the mind is diverted from the subject proper. There should be a perceptible interval between every word and every syllable. Three clergymen out of four fail to meet the requirements of the case; they neither know how to read a chapter or to make an address with correctness and distinctness. The most eminent and successful clergymen of the age—men who "win souls"—have, to a remarkable degree, the gift of a sharp, clear, distinct enunciation ; with this a man may speak in a conversational tone and be heard by five thousand people, where a mumbler will not be heard fifty feet away, although he may have a "thundering" voice. But one minister in five in the City of New York can be comfortably heard in all that he says fifty feet away from his person ; even at that distance many words have to be guessed at, either from the mumbling habit or from the grievous fault of lowering the voice at the end of long words, and at the end of every sentence, the last word of which, although a monosyllable, should be uttered with as much distinctness as any other.

On a Sunday afternoon the Dean of Canterbury preached in Grace Church, New York, to a packed assembly; every seat and every standing point was occupied, and yet, within forty feet of him, whole sentences were uttered without the possibility of distinguishing a single word. There are not six clergymen in the whole City of New York who are more than half heard in the seats farthest from them, and it is a great cruelty to those whose hearing is not young. This is a very important reason why so much excellent preaching it little more than "water spilled on the ground." Within a month of this writing we have heard Spurgeon several times; he does not speak loud by any means, and yet his clear, silvery, soft voice is heard by seven thousand people at a time, but there is scarcely a minister in New York who can be heard by two thousand people at once. It would horrify any one of them to have his sermon read and two or three words left out of every sentence. Generally it is only the middle of sentences that can be heard. One of the best rules is so to enunciate every word and syllable that it shall fall clearly and sharply defined on your own ear, and be equally concerned that the first and last words of a sentence should be clearly heard.

